# Colonization with multidrug-resistant bacteria increases the risk of complications and a fatal outcome after allogeneic hematopoietic cell transplantation

**DOI:** 10.1007/s00277-017-3205-5

**Published:** 2017-12-19

**Authors:** Alicja Sadowska-Klasa, Agnieszka Piekarska, Witold Prejzner, Maria Bieniaszewska, Andrzej Hellmann

**Affiliations:** Department of Hematology and Transplantology, University Clinical Center, Medical University of Gdansk, Dębinki 7, 80-952 Gdańsk, Poland

**Keywords:** Colonization, Infections, Transplantation, Metronidazole, Microbiome

## Abstract

Composition of the gut microbiota seems to influence early complications of allogeneic hematopoietic cell transplantation (HCT) such as bacterial infections and acute graft-versus-host disease (GVHD). In this study, we assessed the impact of colonization with multidrug-resistant bacteria (MDRB) prior to HCT and the use of antibiotics against anaerobic bacteria on the outcomes of HCT. We retrospectively analyzed the data of 120 patients who underwent HCT for hematologic disorders between 2012 and 2014. Fifty-one (42.5%) patients were colonized with MDRB and 39 (32.5%) had infections caused by MDRB. Prior colonization was significantly correlated with MDRB infections (*P* < 0.001), especially bacteremia (*P* = 0.038). A higher incidence of MDRB infections was observed in patients with acute (*P* = 0.014) or chronic (*P* = 0.002) GVHD and in patients aged > 40 years (*P* = 0.002). Colonization had a negative impact on overall survival (OS) after HCT (64 vs. 47% at 24 months; *P* = 0.034) and infection-associated mortality (*P* < 0.001). Use of metronidazole was correlated with an increased incidence of acute GVHD (*P* < 0.001) and lower OS (*P* = 0.002). Patients colonized with MDRB are more susceptible to life-threatening infections. Colonization with virulent flora is the most probable source of neutropenic infection; therefore, information about prior positive colonization should be crucial for the selection of empiric antibiotic therapy. The use of metronidazole, affecting the biodiversity of the intestinal microbiome, seems to have a significant impact on OS and acute GVHD.

## Introduction

Allogeneic hematopoietic cell transplantation (HCT) is the curative option for various hematologic disorders. Despite progress in diagnostic and transplant-related procedures, HCT carries a risk of fatal complications related to graft-versus-host disease (GVHD) and infections [1]. As a result of prolonged neutropenia and disruption of anatomic barriers as well as cellular and humoral immunodeficiency, bacterial infections are common in the early phase after HCT [2]. Mucosal toxicity caused by the conditioning regimens, leading to increased permeability of the gastrointestinal (GI) tract, enables colonizing bacteria to translocate to the circulation and cause bloodstream infections. Moreover, increased exposure of donor-derived lymphocytes to recipient antigens can stimulate the immune system and contribute to the development of alloreactivity [3].

The intestinal microbiome influences the maturation of the immune system and immune-mediated responses. The loss of gut microbiome diversity and generation of multidrug-resistant bacteria (MDRB) are direct consequences of increased consumption of broad-spectrum antibiotics [4–6]. Treatment with antibiotics against anaerobic bacteria, such as metronidazole, can promote proinflammatory responses through the unselective destruction of gut Clostridiales. An imbalance in gut microbiome composition with dominance of MDRB may have an impact on GVHD development and lead to infectious complications. Therefore, we performed a retrospective analysis of the 120 patients to assess the influence of colonization with alert pathogens prior to the transplant procedure and the use of antibiotics against anaerobic bacteria on the outcomes of HCT.

## Patients and methods

This study included all patients who underwent allogeneic HCT at the Department of Hematology and Transplantology, Medical University of Gdansk, Gdansk, Poland, between January 2012 and December 2014. Local Human Research Ethics Committee approved publication of the retrospective analysis since patient-identifying data were omitted to protect anonymity and the microbiological samples were collected as routine tests with prior informed consents of the patients, available in the patients’ medical records.

### Patient characteristics

The study included 120 patients (70 male and 50 female), with a median age of 41 years (range 19–67 years), diagnosed with acute myeloid leukemia or myelodysplastic syndrome (*n* = 65), acute lymphoblastic leukemia (*n* = 25), chronic myeloproliferative neoplasms (*n* = 14), lymphomas or chronic lymphoproliferative neoplasms (*n* = 10), severe aplastic anemia, and/or paroxysmal nocturnal hemoglobinuria (*n* = 6).

Fifty-two patients received hematopoietic cells from matched unrelated donors (MUDs) or mismatched unrelated donors (MMUDs), and 68 received transplants from matched sibling donors. The source of stem cells was peripheral blood was in 111 patients (92.5%) and bone marrow in nine patients (7.5%). Reduced intensity/toxicity conditioning was applied in 24 patients, myeloablative conditioning was used in 92 patients and four patients received immunoablation. In all patients, cyclosporine A and short-term methotrexate were administered as GVHD prophylaxis. The patients who received allotransplants from MUDs/MMUDs additionally received rabbit anti-thymocyte globulin. The characteristics of the study group are presented in Table 1.

**Table 1:** Patient characteristics and colonization status

	All patients	Noncolonized	Colonized
Basic demographic characteristics			
Group size, no. (%)	120 (100)	69 (57.5)	51 (42.5)
Sex distribution: female/male, no. (%)	50 (42)/70 (58)	29 (42)/40 (58)	21(41)/30 (59)
Age at HCT, median (range), years	41 (19–67)	38 (19–67)	44 (21–66)
Age > 40 years, no. (%)	59 (49)	39 (56.5)	20 (39)
Diagnosis			
AML/MDS, no. (%)	65 (54)	39 (57)	26 (51)
ALL, no. (%)	25 (21)	13 (19)	12 (23)
CML/MF/CMML, no. (%)	14 (11.5)	9 (13)	5 (10)
NHL/HL/CLL/MM, no. (%)	10 (8.5)	6 (9)	4 (8)
sAA/PNH, no. (%)	6 (5)	2 (3)	4 (8)
Transplant characteristics			
Donor type: MUD or MMUD/MSD, no. (%)	68 (57)/52(43)	40 (58)/29 (42)	28 (55)/23 (45)
Graft source: PB/BM, no. (%)	111 (92.5)/9 (7.5)	64 (93)/5 (7)	47 (92)/4 (8)
Conditioning regimen: MAC/RTC or RIC, no. (%)	92 (77)/24 (20)	55 (80)/12 (17)	37 (72.5)/12 (23.5)
Conditioning regimen: immunoablation, no. (%)	4 (3)	2 (3)	2 (4)
Day of neutrophil engraftment, median (range)	23 (14-not achieved)	23 (14-not achieved)	23 (14-not achieved)
ANC > 500/mm^3^ before day 20, no. (%)	36 (30)	24 (35)	12 (24)

*HCT* hematopoietic cell transplantation, *AML*/*MDS* acute myeloid leukemia/myelodysplastic syndrome, *ALL* acute lymphoblastic leukemia, *CML* chronic myeloid leukemia, *MF* myelofibrosis, *CMML* chronic myelomonocytic leukemia, *NHL* non-Hodgkin lymphoma, *HL* Hodgkin lymphoma, *CLL* chronic lymphocytic leukemia, *MM* multiple myeloma, *sAA* severe aplastic anemia, *PNH* paroxysmal nocturnal hemoglobinuria, *MUD* matched unrelated donor, *MMUD* mismatched unrelated donor, *MSD* matched sibling donor, *MAC* myeloablative conditioning, *RTC* reduced toxicity conditioning, *RIC* reduced intensity conditioning, *ANC* absolute neutrophil count

### Anti-infectious prophylaxis

Transplantation-related procedures were performed according to institutional protocols. All patients were placed in single rooms with increased sanitary requirements (contact isolation, high-efficiency particulate air [HEPA] filters) and fed a neutropenic diet. Every patient received ciprofloxacin, acyclovir, and an anti-fungal agent as standard anti-infective prophylaxis.

### Colonization

Rectal and nasal swabs and stool specimens were collected upon admission to the hospital and routinely repeated on a weekly basis during hospitalization. Colonization was defined as positive in the case of culture growth from at least one swab or a stool probe. The alert pathogens included: vancomycin-resistant *Enterococcus* (VRE), extended-spectrum beta-lactamase (ESBL)-producing pathogens, and carbapenem-resistant *Pseudomonas aeruginosa* (CRPA). Carbapenem-resistant *Acinetobacter baumannii* and methicillin-resistant *Staphylococcus aureus* were not detected in the study group.

### Statistical analysis

Categoric variables were expressed as absolute numbers and respective percentages, and the differences between groups were compared using Pearson’s χ^2^ test. Continuous variables were expressed as median values with ranges. The relationship between continuous and categoric variables was analyzed using a nonparametric Mann–Whitney *U* test. Survival analysis was performed according to the Kaplan–Meier method. Overall survival (OS) was calculated from the date of transplantation until death from any cause. The study population was stratified according to principal clinical and demographic characteristics, and the mean values of the groups were compared using the log-rank test. Multivariate Cox regression analysis was applied to identify independent predictive factors. A *P* value of < 0.05 was considered statistically significant. All analyses were performed using STATISTICA version 12 (StatSoft, Inc., Tulsa, OK, USA).

## Results

### Colonization and infections

Colonization with MDRB was detected in 42.5% of the patients. The most common colonizing MDRB were the following: VRE (39%), ESBL-producing *Escherichia coli* (27%), ESBL-producing *Klebsiella pneumoniae* (20%), and CRPA (5%). Multiple colonizing alert pathogens were cultured from 14% of the patients.

Despite antimicrobial prophylaxis, microbiologically documented bacterial infections occurred in 91% and fever of unknown origin was observed in 72.5% of the patients. MDRB infections were diagnosed in 32.5% of the entire study group (39 patients). Among the patients who developed MDRB infections, 50% occurred to be colonized upon admission to the transplant unit and the rest of them acquired the positive colonization status during the hospitalization. There was a significantly higher incidence of MDRB infections among the group previously colonized (*P* < 0.001), and the pathogen most commonly detected was ESBL-producing *K. pneumoniae* (31%).

**Fig. 1:**
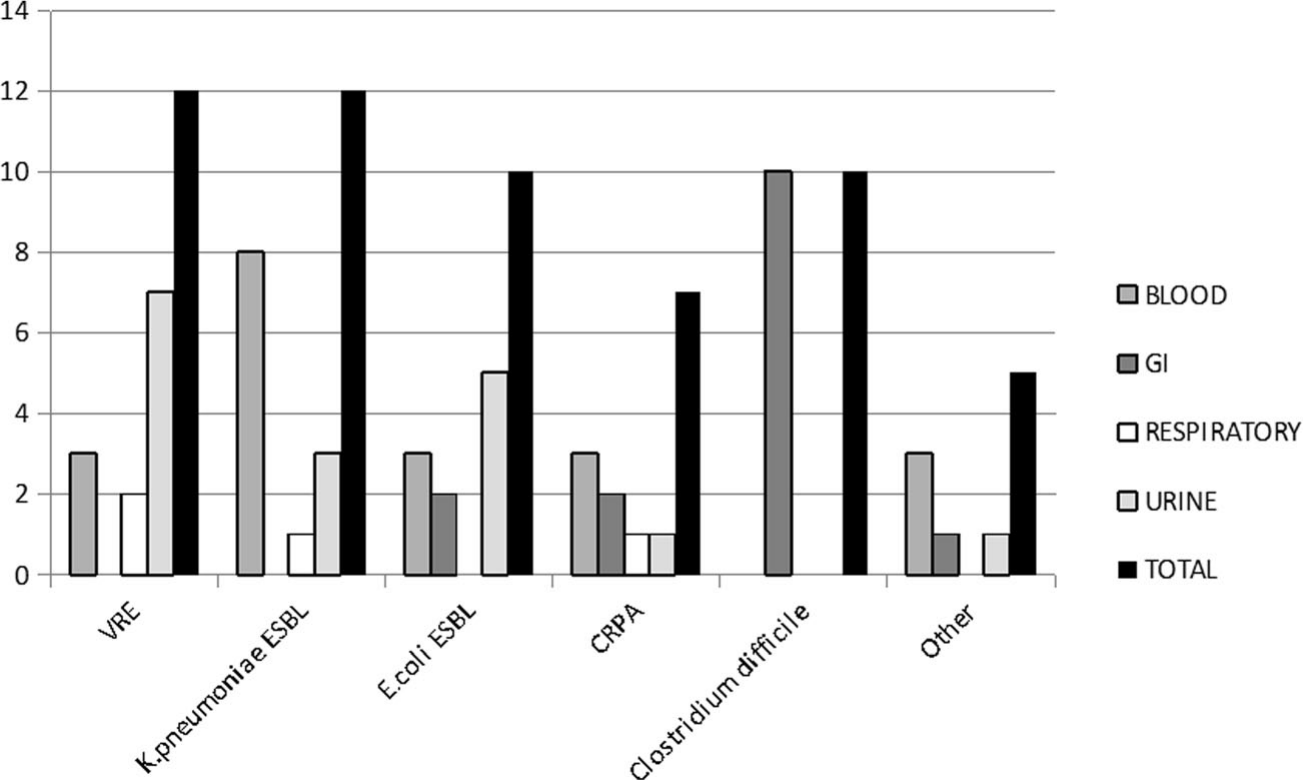
Distribution of etiological factors and types of infection. Vancomycin-resistant *Enterococcus* (VRE), extended-spectrum beta-lactamase (ESBL)-producing *Klebsiella pneumoniae* (*K. pneumoniae* ESBL), ESBL-producing *Escherichia coli* (E.coli ESBL), carbapenem-resistant *Pseudomonas aeruginosa* (CRPA)

GI infections were diagnosed on the basis of symptomatic enterocolitis and the isolation of any bacteria potentially pathogenic to the intestines (VRE and *Klebsiella* isolates were excluded). There were 16 (41%) urinary tract infections, 13 (33.8%) bloodstream infections, 6 (15.4%) GI infections, and 4 (10.3%) respiratory tract infections. The distribution of the etiologic factors and types of infection are presented in Fig. 1. *Clostridium difficile* enterocolitis was added to the graphic presentation as a direct consequence of antibiotic therapy.

There was a significantly higher incidence of MDRB bacteremia in the colonized group than in the noncolonized group (16 vs. 6%; *P* = 0.038), and ESBL-producing *K. pneumoniae* was the most common cause of bloodstream infections (69%). There was a significant correlation between pre-colonization with ESBL-producing *K. pneumoniae* (*P* < 0.001), ESBL-producing *E. coli* (*P* = 0.01), and CRPA (*P* = 0.003) and subsequent bacteremia caused by one of these pathogens. However, we did not observe a significant correlation in the case of colonization with VRE (*P* = 0.08).

### Engraftment, graft-versus-host disease, and infections

The median time between the detected positive MDRB colonization and infection occurrence was 16 days (mean 26 days). Most infections developed during agranulocytosis but there was the second peak in patients treated due to GVHD. The median time to engraftment was 23 days after HCT. Early regeneration of hematopoiesis, defined as an absolute neutrophil count ≥ 500/mm^3^ before day 20 after HCT, was observed in 36 patients. Ten patients did not achieve engraftment. There was no significant correlation between the day of engraftment and the incidence of infections with alert pathogens (*P* = 0.25). MDRB infections were more common in patients with active acute (*P* = 0.014) or chronic GVHD (*P* = 0.002), as well as in patients age > 40 years (*P* = 0.002).

### Colonization and graft-versus-host disease

Acute GVHD was diagnosed in 22% of the patients (grade I/II, 9%; grade III/IV, 13%), with subsequent chronic GVHD development in 14% of the patients. The incidence of acute GVHD showed a tendency to be higher in the colonized group than in the noncolonized group (27 vs. 18%); however, the difference was not significant (*P* = 0.26). We observed a higher incidence of acute GVHD among patients colonized with more than one species of MDRB (*P* = 0.046).

### Metronidazole and acute graft-versus-host disease

Of the study group, 17 patients (14%) received metronidazole, as treatment for *C. difficile* enterocolitis in ten patients and for other indications (cholecystitis, *Helicobacter pylori* eradication, typhlitis) in seven patients. *C. difficile* was the cause of infections in 13 HCT recipients (Fig. 1). Metronidazole was the first therapeutic choice in 10 patients (77%); however, only three patients responded, and the remaining seven patients required vancomycin for persistent infection. Three of the 13 patients received vancomycin as the first-line treatment and experienced complete resolution of the symptoms.

A sub-analysis of the proportion of our study group treated with broad-spectrum antibiotics against anaerobic bacteria revealed a temporal relationship between the introduction of metronidazole for *C. difficile* infection and the development or aggravation of acute GVHD (*P* < 0.001). A similar correlation was noted in the patient population treated with metronidazole for reasons other than *C. difficile* infection (*P* < 0.05).

### Mortality

**Fig. 2:**
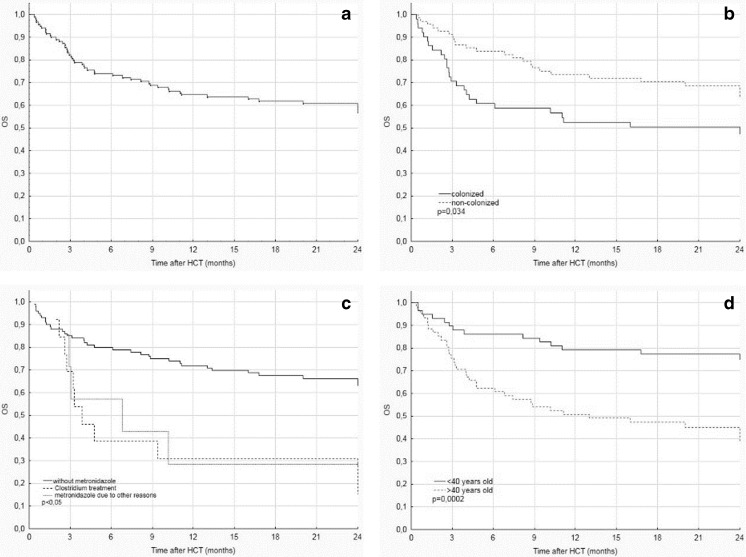
Factors influencing overall survival after hematopoietic cell transplantation. **a** Overall survival (OS) in the entire study group; **b** effect of colonization with multidrug-resistant bacteria (solid line) versus no prior colonization (dashed line); **c** OS after metronidazole use for *Clostridium difficile* infection (dashed line) and other indications (dotted line) versus OS in patients unexposed to metronidazole (solid line); **d** OS stratified by age < 40 (solid line) versus > 40 (dashed line) years. Overall survival (OS), hematopoietic cell transplantation (HCT)

The median follow-up time was 17 months (range 0.4–47 months). The estimated OS rates for the entire study group were 73, 65, and 59% after 6, 12, and 24 months post-HCT, respectively (Fig. 2a). Forty-nine patients (41%) died during follow-up from the following: relapse (13%), infections (10%), GVHD-related complications (14%), or both GVHD and infections (3%).

Analysis of the MDRB-colonized group showed a significantly higher mortality rate (*P* = 0.034) and infection-associated mortality rate (*P* < 0.001) compared with the noncolonized group. The OS rates at 24 months after HCT were 47 and 64% in the colonized and noncolonized groups, respectively (*P* < 0.05; Fig. 2b). Multivariate analysis identified HCT from MUDs (hazard ratio [HR], 2.2; 95% confidence interval [CI], 1.2–4; *P* = 0.01) and metronidazole use (HR, 3; 95% CI, 1.6–5.9; *P* = 0.001) as factors associated with reduced OS (Fig. 2c). Age < 40 years at transplantation was associated with a better OS (HR, 0.3; 95% CI, 0.2–0.6; *P* = 0.0002; Fig. 2d).

## Discussion

Despite progress in diagnosis and therapy, infections and GVHD-related complications remain the most common causes of transplant-related mortality (TRM) post-HCT [1]. Eradication of the gut microbiome has been a subject of interest since the early 1970s; at the time, a germ-free environment was believed to limit infection rates and GVHD development [7, 8]. Later, isolation procedures, HEPA filters, a neutropenic diet, and gut decontamination using antibiotics were introduced to reduce TRM [9–12]. Now, taking into account the immunoregulatory role of the healthy microbiome, the routine administration of prophylactic antibiotics in hemato-oncological patients and after HCT should be reconsidered [13–16].

The burden of colonizing bacterial flora was recently estimated to be about 0.2 kg, and the number of bacterial cells in the human body was determined to be comparable to that of host cells [17]. The composition of the gut microbiome can be precisely assessed, and > 1000 species have already been identified by 16 ribosomal RNA gene sequencing [18]. Besides the degradation of digestion products and production of vitamins, the intestinal microbiome plays a pivotal role in immune system regulation. It maintains the balance between the pro- and anti-inflammatory effectors, including immunotolerant regulatory T lymphocytes (Tregs) and proinflammatory Th17 lymphocytes [19–26]. Th17 lymphocytes protect the intestinal mucous membrane from pathogens but, in certain circumstances, can catalyze an inflammatory process that leads to GVHD development [27]. In the presence of short-chain fatty acids produced by Clostridiales, naïve T lymphocytes are induced for the generation of extrathymic Tregs [24, 28, 29], whereas proinflammatory cytokines or segmented filamentous bacteria direct them to generate Th17 cells [30]. Intestinal epithelial cells (IECs) create a physiologic and biochemical barrier between the commensal microorganisms of the gut and host tissues [21]. High-dose chemo- and radiotherapy prior to HCT impairs GI epithelial integrity, which may aid the translocation of colonizing bacteria into the circulation, resulting in severe infectious complications. Additionally, increased exposure to antigens of the host histocompatibility complex related to IECs damage promotes acute GVHD development [3, 31].

In the study group, 42.5% of the patients were colonized with MDRB and 14% were carriers of more than one alert pathogen. The high percentage of colonized patients is unsurprising: many of the patients requiring HCT had a history of aggressive treatment of the underlying disease, requiring prolonged hospitalization and administration of broad-spectrum antibiotics for life-threatening infections. Colonization with MDRB had a significant impact on non-relapse mortality, leading to a lower OS 2 years after HCT and higher mortality due to infections than those of noncolonized patients. The higher mortality rate in the colonized group may have been caused by the loss of microbiome diversity and consequent expansion of pathogenic bacteria within the niches of gut bacteria [32–34]. Bloodstream infections with MDRB were more common in patients colonized with ESBL-producing *K. pneumoniae*, ESBL-producing *E. coli*, and CRPA. There was no significant correlation between colonization with VRE and bacteremia caused by this pathogen. Similar results have been presented by other authors [35, 36]. During episodes of neutropenic fever, the etiological factor is not always identified. Because the colonizing virulent flora is the most probable source of a neutropenic infection, information about prior positive colonization is important for the selection of empiric antibiotic therapy to reduce the risk of a fatal outcome.

As mentioned earlier, the fragile balance between pro- and anti-inflammatory mechanisms can be disrupted by changes in microbiome composition, resulting from conditioning chemotherapy and the use of antibiotics. In murine models, disturbances in the commensal gut flora, with domination of Enterobacteriales (*E. coli*, *Klebsiella*, and *Enterobacter*), Lactobacillales (*Lactobacillus*, *Enterococcus*, and *Streptococcus*), and a reduction in Firmicutes species (including Clostridiales), were correlated with acute GVHD development [37]. A similar shift towards enterococci, particularly observed after antibiotic prophylaxis and confirmed by the metagenomic analysis of the stool microbiome, preceded the intestinal manifestation of acute GVHD in a human population [38]. This phenomenon can be explained by the ability of enterococci to create a biofilm and produce epitheliolysins and other toxins that disrupt the integrity of the epithelial barrier, intensifying inflammatory and immune responses and leading to increased production of proinflammatory substances, such as tumor necrosis factor [39, 40]. Although we did not find a significant correlation between colonization with VRE and the occurrence of acute GVHD or VRE bacteremia, acute GVHD was more common in the group colonized with multiple alert pathogens, including VRE.

Enterococci have the ability to grow excessively in favorable conditions, pushing the commensal flora out of their niches. Although prophylactic use of ciprofloxacin lowers the rate of Gram-negative bacteremia, it does not prevent streptococcal or enterococcal septic episodes and, by causing an imbalance in anaerobic commensals, may increase the risk of colonization with MDRB, such as VRE [41]. This observation is confirmed by the profile of colonizing bacteria in the proportion of our study group receiving ciprofloxacin prophylaxis; despite prophylaxis, > 90% of the patients developed infectious complications during the neutropenic period after HCT. In contrast to fluoroquinolones, rifaximin represents a perfect prophylactic agent that provides protection against bacteremia and preserves the physiologic balance of the gut microbiome [42, 43].

Of the intestinal microbiota, 90% consists of anaerobic bacteria [44]. Administration of broad-spectrum agents selectively targeting anaerobic bacterial flora may lead to excessive proliferation of aerobic and relatively anaerobic pathogens. Among the HCT recipients examined, patients treated with metronidazole exhibited a significantly lower OS. Moreover, the incidence of acute GVHD with more severe manifestation (grade III/IV) was higher in the metronidazole-treated group. This observation may be partially related to the unselective depletion of all Clostridiales, alongside the desired elimination of *C. difficile*. Therapy with metronidazole failed in most patients with *C. difficile* infections. Based on our data and the guidelines of the European Society of Clinical Microbiology and Infectious Diseases, patients with *C. difficile* infections after HCT should be treated as a high-risk group for severe complications. Therefore, more potent oral vancomycin or novel therapies (e.g., fidaxomicin) should be administered instead of metronidazole as the first-line therapy [45]. The impact of the microbiome on many conditions, including autoimmune disorders [19, 20, 46, 47], cancer [48], and chemo-resistance [49], indicate the necessity of new strategies that maintain the physiologic composition of the gut microbiota. Fecal microbiome transplantation (FMT) has been investigated as a method to restore the composition of the gut microflora, and may eradicate MDRB before HCT, leading to reduction in acute GVHD and TRM. The first data concerning the successful use of FMT for steroid-resistant gut acute GVHD, *C. difficile* infection, or decolonization of resistant pathogens have been already published [50, 51].

Homeostasis of the human microbiome is important and requires further investigation. The data already available, confirmed by the findings of our study, have practical implications for the selection of prophylactic and infection-driven antibiotic strategies that may improve the outcomes of immunocompromised patients after HCT.
